# Localization of the Dual Oxidase BLI-3 and Characterization of Its NADPH Oxidase Domain during Infection of *Caenorhabditis elegans*

**DOI:** 10.1371/journal.pone.0124091

**Published:** 2015-04-24

**Authors:** Ransome van der Hoeven, Melissa R. Cruz, Violeta Chávez, Danielle A. Garsin

**Affiliations:** Department of Microbiology and Molecular Genetics, The University of Texas Health Science Center at Houston, TX 77030, United States of America; University of Rochester Medical Center, UNITED STATES

## Abstract

Dual oxidases (DUOX) are enzymes that contain an NADPH oxidase domain that produces hydrogen peroxide (H_2_O_2_) and a peroxidase domain that can utilize H_2_O_2_ to carry out a variety of reactions. The model organism *Caenorhabditis elegans* produces the DUOX, BLI-3, which has roles in both cuticle development and in protection against infection. In previous work, we demonstrated that while certain peroxidases were protective against the human bacterial pathogen *Enterococcus faecalis*, the peroxidase domain of BLI-3 was not, leading to the postulate that the NADPH oxidase domain is the basis for BLI-3’s protective effects. In this work, we show that a strain carrying a mutation in the NADPH oxidase domain of BLI-3, *bli-3(im10)*, is more susceptible to *E*. *faecalis* and the human fungal pathogen *Candida albicans*. Additionally, less H_2_O_2_ is produced in response to pathogen using both an established Amplex Red assay and a strain of *C*. *albicans*, WT-OXYellow, which acts as a biosensor of reactive oxygen species (ROS). Finally, a *C*. *elegans* line containing a BLI-3::mCherry transgene was generated. Previous work suggested that BLI-3 is produced in the hypodermis and the intestine. Expression of the transgene was observed in both these tissues, and additionally in the pharynx. The amount and pattern of localization of BLI-3 did not change in response to pathogen exposure.

## Introduction

Understanding the innate immunity of epithelial surfaces is crucial for protecting against agents of infectious disease and inflammatory immune pathologies. One innate immune mechanism is ROS (reactive oxygen species) production by NADPH oxidases of the NOX/DUOX (NADPH oxidase/dual oxidase) family. NOX enzymes consist of one NADPH oxidase domain and most often produce O_2_^-^ (superoxide). DUOX enzymes have an additional peroxidase domain, and the NADPH oxidase domain is characterized as generating H_2_O_2_ (hydrogen peroxide) [1,2]. DUOX enzymes are often found in epithelial tissue. For example, human DUOX mRNA is found in the salivary glands and both the airway and gastrointestinal epithelium [3,4] where it is postulated to play a role in both physiological and pathological immune responses.

The role and function of DUOX in host defense has been modeled in a variety of organisms including the worm (*Caenorhabditis elegans*) the zebrafish (*Danio rerio*) and the fly *(Drosophila melanogaster*), where loss of the enzymes causes increased susceptibility to microbial infection [5–7]. Mammals encode five NOX and two DUOX enzymes that are active in a variety of tissues and cellular compartments [1,2]. Such complexity can make identifying the source and effects of ROS generated from NADPH oxidases difficult at the organismal level. Among animals in which genes encoding NADPH oxidases have been identified, *C*. *elegans* is unique in that it only produces one functional NADPH oxidase, the DUOX, BLI-3 [8,9]. Technically, it harbors the sequence for a second gene, an apparent duplication of *bli-3*, but a gene product does not appear to be produced [8] and our studies indicate that a deletion of the gene results in no observable phenotypes [5]. *C*. *elegans’* unique feature of producing a single DUOX enzyme allows us to study the roles of DUOX-generated H_2_O_2_ during infection in the natural context of the whole organism without having to account for the effects of other NADPH oxidases.

In previous work, we showed by partial RNAi knock-down and by using a chemical inhibitor of NADPH oxidases, diphenyliodonium chloride (DPI), that BLI-3 is the source of ROS released during infection with the human pathogen *Enterococcus faecalis* [5,10]. We additionally showed that loss of BLI-3 increased susceptibility to *E*. *faecalis* [5]. One technical complication to studying BLI-3’s contribution to innate immunity is its crucial role in development. The H_2_O_2_ generated by the NADPH oxidase domain of BLI-3 is utilized by the peroxidase domain to produce tyrosyl radicals in certain collagen components [8]. A separate peroxidase, MLT-7, also contributes to this process [11]. These highly reactive groups then react with one another to create di- and tri-tyrosine cross-links between the collagens, resulting in the structured outer layer that is the cuticle of the worm [8]. In fact the *bli-3* gene was originally named for the “blistered” phenotype it causes in animals that have point mutations in the peroxidase domain that leads to loss of cuticle integrity [12,13]. While these peroxidase domain point mutants are still viable, probably due to the contribution of MLT-7, complete loss of BLI-3 is not compatible with life.

Interestingly, we did not observe less ROS production or increased susceptibility when the peroxidase point mutants were exposed to *E*. *faecalis*, despite the blistered phenotype, leading us to believe that only the NADPH oxidase domain was required for protection against this pathogen [5]. Also, we discovered a role for additional, separate peroxidases [14], which supports our postulate that the NADPH oxidase domain of BLI-3 plays a role in immunity against *E*. *faecalis*, but not the peroxidase domain. In contrast, Jain et al. did observe some role for the peroxidase domain of BLI-3 in ROS production when animals were exposed to *Saccharomyces cerevisae* [15]. It may be that the peroxidase domain mutation negatively affects the H_2_O_2_ output of the NADPH oxidase domain under some conditions.

Recently, a new *bli-3* point mutation in the region encoding the NADPH oxidase domain was isolated [16]. In a heterologous cell system, the resulting gene product was defective for ROS production, but the *C*. *elegans* line containing this mutation was still viable [16]. We have obtained this mutant to further test our model that the NADPH oxidase domain is required for ROS production and protection against pathogen. Additionally, we have developed a new assay to assess ROS production in the worm using a biosensor strain of the human fungal pathogen, *Candida albicans* [17]. Finally, previous work suggested that BLI-3 is present in the intestine and the hypodermis [5,8], but we further examined tissue localization by generating a fusion to mCherry and discovered that it is additionally present in the pharynx.

## Materials and Methods

### Strains

The following bacterial strains were used in this study: *E*. *coli* OP50 [12], *E*. *faecalis* OG1RF [18], *C*. *albicans* SC5314 [19] and WT-OXYellow [17].

*C*. *elegans* strains were grown and maintained as previously described [20]. The Bristol wild type N2 strain and *bli-3(e767)* were obtained from The Caenorhabditis Genetics Center (University of Minnesota) [12]. *bli-3(im10)* was generously provided by Dr. Moribe [16].

### Amplex Red Assays

The Amplex Red Kit (Invitrogen) assay was previously adapted to *C*. *elegans* to measure pathogen-stimulated H_2_O_2_ release [5,10]. The same protocol was followed with the following modifications. L3 worms were exposed to *E*. *faecalis* for 16 hours. Rather than absorbance, the fluorescence of the buffer containing the Amplex Red reagent and 30 worms was measured after 30 minutes of incubation (540/590nm excitation and emission, respectively). The experiments were performed in triplicate per condition for a total of about 90 worms assayed. The statistical differences between conditions were determined by an unpaired t-test. GraphPad Prism 5.0 (GraphPad Software, San Diego, CA) was used for the analysis.

### Fluorescence Microscopy

After L3 stage worms were infected with *C*. *albicans* WT-OXYellow [17] and allowed to incubate overnight. They were washed with 1 ml of sterile M9W and collected by centrifugation at 1000 rpm. The wash was repeated three times. The worms were then paralyzed with 1mM tetramisole for 30 minutes. Anesthetized worms were mounted on 2% agarose pads and imaged using an Olympus IX81 automated inverted microscope and Slidebook software (Version 6.0). Imaging and photography was performed using FITC and TRITC filter sets to capture *CTA1-yEGFP* and *ADH1-yCherry* expression, respectively. The FITC and TRITC images were used to quantify expression of CTA1-yEGFP in comparison to ADH1-yCherry to obtain a normalized measurement of ROS production. The amount of fluorescence for each filter set was quantified using ImageJ 1.48 freeware. The fluorescence was measured in the worm intestine within a defined area. The same area of background was subtracted. The ratio of the FITC to the TRITC measurements was calculated. A total of 28–30 worms were measured and averaged for each strain and the standard error was calculated. Statistical differences were determined by an unpaired *t*-test using GraphPad Prism version 5.0 (GraphPad Software, San Diego, CA). Each experimental condition was compared pairwise to the control condition. *P*-values < 0.05 were considered to be statistically significant.

### Killing and Longevity Assays

For all survival assays in this study, the nematodes were rendered sterile by *cdc-25*.*1* RNAi as previously described [21], except that the exposure was begun in the gravid adults that generated the sterile progeny used for our experiments. All survival assays were begun at the L4 stage. Infection with *E*. *faecalis* and *C*. *albicans* was performed as described previously [22–24]. Longevity assays with *E*. *coli* and heat killed *E*. *coli* were also performed as previously described, except that FUDR was not added to the plates because the animals were already sterile [25]. The assays were performed at 25°C and worm survival was scored daily. An n of 60–90 animals was used in for each condition and all experiments were replicated at least three times. GraphPad Prism 5.0 was used for Kaplan-Meier log rank analysis and survival curves were compared pairwise with a *P* < 0.05 considered statistically significant.

### Construction of BLI-3 Transgenic

A 5kb fragment containing the promoter region and first two exons of the *bli-3 gene* was amplified using the forward primer containing a PstI site that is underlined, AAAAAACTGCAGCATAACTTTGCTGAACCTGGTCCGCGGCAG, and the reverse primer, AAAAAAGGATCCGAATAGTATATCGGAGAGTTCACGGGCGGA with the BamHI site underlined. Subsequently, the fragment was cloned into the PstI and BamHI sites of vector pPD95.77 (generated by A. Fire and distributed by Addgene as plasmid 1495) harboring *mcherry* instead of *gfp* and transformed in *E*. *coli* DH5α. The construct was verified by sequencing. 100 μg of the plasmid was injected into the gonads of young well fed gravid hermaphrodites. The worms were allowed to recovery on seeded plates and the resulting progeny were observed for mCherry expression using a fluorescent dissecting microscope. Four independent extra-chromosomal lines were obtained. The expression of mCherry was similar in all four lines. Two of the lines were exposed to Trimethylpsoralen (TMP), UV irradiated and backcrossed four times to select for stable, integrated transgenic worms.

### Western Blotting

Worms were collected after exposure to either *E*. *coli* or *E*. *faecalis* for 18 hours. A 100μl worm pellet was collected from about 6–8 NG plates per condition. The pellet was washed with 500μl of 500X protease inhibitors (Roche) mixed with TEGN (20mM Tris-HCl (pH = 7.9), 0.5 mM EDTA, 10% glycerol, 50mM NaCl) at 2μl per 1mL respectively. The pellet was disrupted using a mortar and pestle in microcentrifuge tubes. The pellet was resuspended in 100μl TEGN/protease inhibitors and treated to determine whole cell lysate protein concentrations. 2X concentrated solubilizing buffer was added and boiled for 5 minutes. The pellet was spun down at maximum speed for one minute, supernatant was removed and placed in a new centrifuge tube. Samples were electrophoresed through a 6% polyacrylamide gel for BLI-3 protein and a 10% gel for the α-Tubulin control. The gel was transferred to nitrocellulose using electrophoresis at 4°C for 45 minutes. Nitrocellulose was blocked in 5% milk/TBST (20 mM Tris-HCl, pH 7.5 150 mM NaCl) for 15 minutes. The blot was incubated while rocking for one hour at room temperature with primary antibodies as follows: anti-BLI-3 polyclonal rabbit serum from the Lambeth lab was used at 1:1000 concentration [8], anti- α -tubulin (SIGMA) was used at a 1:4000 dilution. Each blot was rinsed several times with TBST for 25 minutes. The secondary antibodies anti-mouse and anti-rabbit peroxidase conjugates were exposed to the blot for 30 minutes at room temperature at a 1:5000 dilution. The blots were rinsed several times with TBST for 25 minutes. Blots were developed using a chemiluminescence assay. The experiment was repeated five times. Protein concentrations were quantified using the AlphaEase AlphaImager 2200 on a FluorChem 8800 and the ratios of BLI-3 to Tubulin calculated.

## Results and Discussion

### A lesion in BLI-3’s NADPH oxidase domain reduces pathogen-induced H_2_O_2_ production

**Fig 1:**
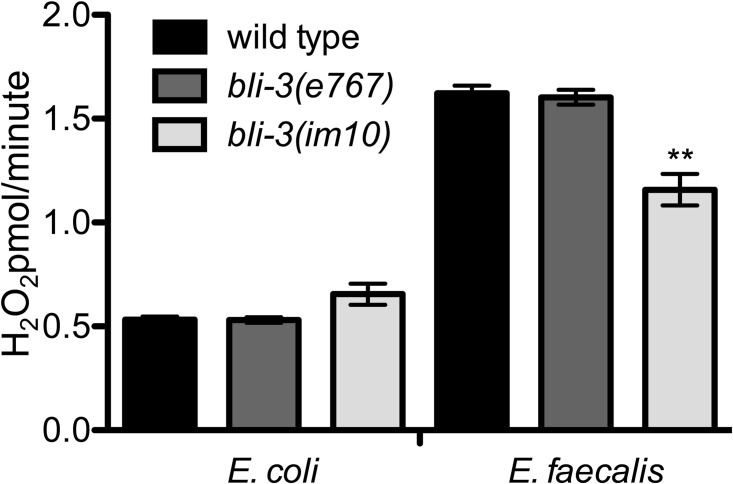
Less H_2_O_2_ production is observed in the *bli-3*(*im10*) mutant in comparison to the wild type worms during infection. H_2_O_2_ production was measured by an Amplex Red assay. While no differences were observed with uninfected animals, significantly less H_2_O_2_ production was observed with infected *bli-3(im10*) mutants compared to infected wild type worms (*P* = 0.0052). The difference between wild type and *bli-3(e767)* was not significant (*P* = 0.7112). The experiment was performed in triplicate and one of two replicates is shown.

Recently, a point mutation in the NADPH oxidase was isolated from a screen for mutant animals with the *bli* phenotype [16]. We obtained this mutant (P1311L, *bli-3(im10)*) and tested it in the modified Amplex Red assay that we and others have used to measure H_2_O_2_ generation in *C*. *elegans* following infection [5,10,15]. As observed in Fig 1, notably less H_2_O_2_ was produced from the *bli-3(im10)* animals compared to N2 wild type animals following infection with *E*. *faecalis*. There was no difference between wild type animals and those with a lesion in the peroxidase domain of BLI-3 (*bli-3(e767)*), in agreement with our previous publication [5].

**Fig 2:**
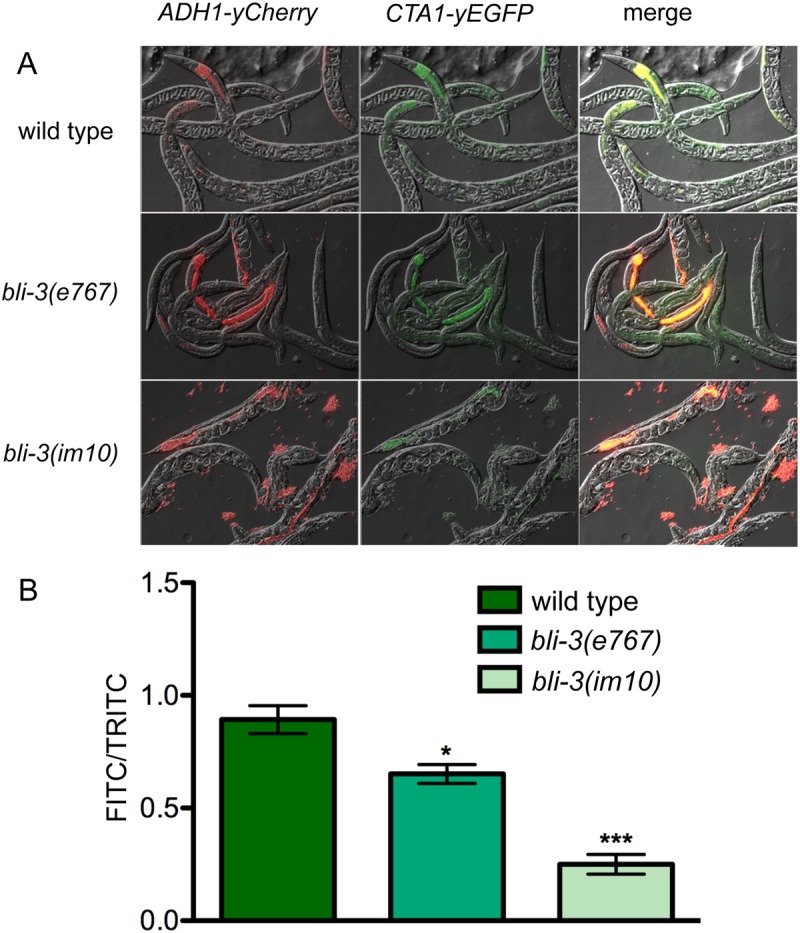
Less *CTA1-yEGFP* expression is observed in the *bli-3* mutants in comparison to the wild type worms during infection with *C*. *albicans* strain WT-OXYellow. **(A)** Following exposure of *C*. *elegans* to WT-OXYellow for 16 hours, fluorescent pictures were taken of animals under the appropriate filters to capture both the *ADH1-yCherry* (TRITC) and the *CTA1-yEGFP* (FITC) markers. These were merged with Nomarski views. Finally, the two markers were merged to compare their relative strengths. Representative pictures of the *bli-3(im10)*, *bli-3(e767)* and wild type are shown. **(B)** From the pictures taken in (**A**), the expression levels of the *CTA1-yEGFP* (FITC channel) relative to *ADH1-yCherry* (TRITC) were quantified as described in Materials and Methods. N = 28–30 worms for each condition. * indicates a statistically significant difference in comparison to wild type (*P* = 0.0243), whereas *** indicates a difference of *P* < 0.0001.

To examine the response to a fungal pathogen and provide an independent means by which to measure H_2_O_2_ generation by the worm, we utilized a strain of the human fungal pathogen *Candida albicans*, called WT-OXYellow, essentially as a biosensor. WT-OXYellow was previously utilized to detect oxidative stress in zebrafish. It contains *yEGFP* (yeast codon-optimized enhanced GFP) fused to the promoter of the gene that encodes for catalase, *CTA1* [17]. A similar *CTA1-GFP* transgenic strain was shown to generate GFP in response to exogenously added H_2_O_2_ and in oxidative environments such as the phagocytes of innate immune cells, but not in response to other stresses such as heat, osmotic or nitrosative stress [26]. WT-OXYellow also contains a fusion of *yCherry* (yeast codon optimized mCherry) to the promoter of *ADH1*, a constitutively expressed gene encoding alcohol dehydrogenase [17]. *C*. *elegans* were infected with this strain of *C*. *albicans* in a manner similar to that described in previous work [22,23]. After a 16-hour incubation following infection with *C*. *albicans*, the animals were washed, placed on agarose pads, and viewed under the fluorescent microscope. Pictures were taken using appropriate filters to detect the fluorescent markers and representative examples are shown if Fig 2A. From these pictures, the amount of *yCherry* and *yEGFP* expression was measured and the ratio between the two determined (Fig 2B). As shown in Fig 2, animals with a lesion in the peroxidase domain of BLI-3 *(bli-3(e767)*) had slightly less *yEGFP* expression (relative to *yCherry* expression) compared to the wild type strain. In contrast, mutants with a lesion in the NADPH oxidase domain of BLI-3 (*bli-3(im10)*) displayed significantly less *yEGFP* expression than wild type animals. From these experiments, we conclude that mutation in the oxidase domain caused by the *bli-3(im10)* allele results in less H_2_O_2_ production in response to both *E*. *faecalis* and *C*. *albicans*. Additionally we have shown that the WT-OXYellow *C*. *albicans* strain, originally used in the zebrafish model [17], can also be used in *C*. *elegans* to examine the oxidative environment within the host.

Previous work on wild type and mutant BLI-3 proteins was performed in a heterologous system in which BLI-3 and the necessary accessory factors were produced from stable transfectants in mammalian HT1080 cells [16]. The mutant versions of BLI-3 were shown to be as stable as wild type [16]. However, cells containing wild type *bli-3* produced H_2_O_2_, but not cells containing *bli-3(im10)*, which encodes for the NADPH oxidase domain mutant, mirroring our results in the whole animal (Figs 1 and 2). In contrast, the heterologous system was also completely inactive for H_2_O_2_ production when carrying *bli-3(e767)*, encoding for the mutation in the peroxidase domain [16]. The result is in contrast to assays utilizing intact *C*. *elegans* where activity is not impacted, or only partially, depending on the pathogen (Figs 1 and 2 [5,15]). Therefore, it is possible that the peroxidase domain does contribute to ROS production and the *bli-3(e767)* allele partially retains this function in vivo. Alternatively, another one of the many peroxidases found in *C*. *elegans* could complement the function in terms of immune H_2_O_2_ production [14]. Finally, it is possible that the mutant protein behaves differently depending on whether a trigger activates it—pathogen exposure in our assays vs. the basal level of activity examined in the heterologous system [16].

### A lesion in BLI-3’s NADPH oxidase domain increases sensitivity to pathogen and diminishes lifespan

**Fig 3:**
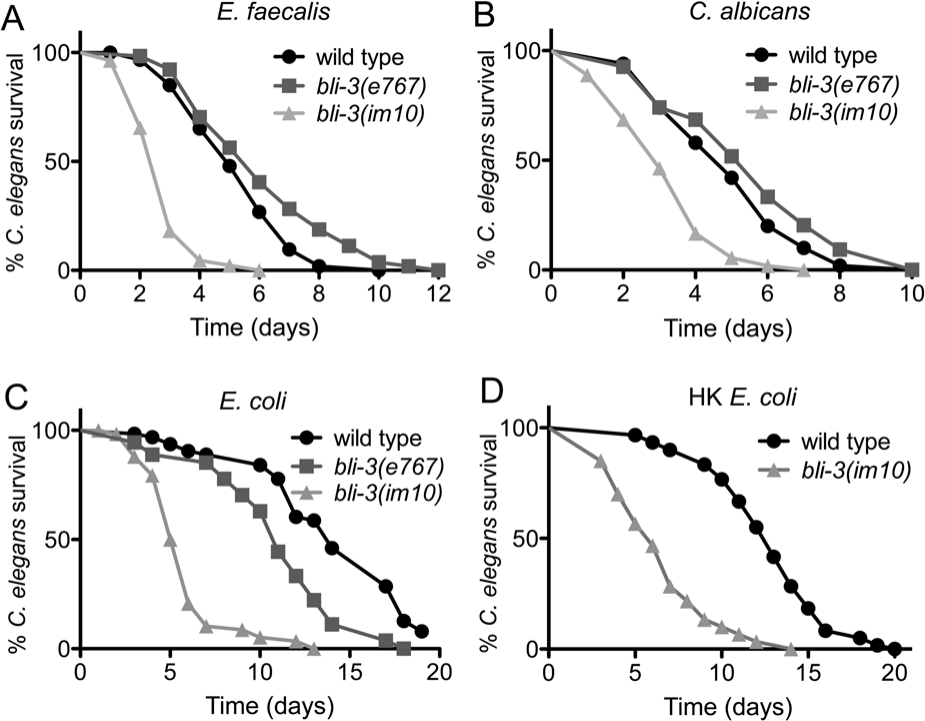
A mutation in the BLI-3 NADPH oxidase domain increases sensitivity to pathogen and shortens lifespan. *bli-3(im10)* mutants exhibited increased susceptibility after exposure to **(A)** *E*. *faecalis*, **(B)** *C*. *albicans* compared to wild type animals (*P* < 0.0001 and *P* = 0.0056 respectively). Differences were not significant between *bli-3(e767)* and wild type. **(C)** *bli-3(im10)* displayed shortened survival in the standard *E*. *coli*-based longevity assay compared to wild type (*P* < 0.0001). *bli-3(e767)* also exhibited a defect in comparison to wild type in this assay (*P* < 0.0001). **(D)** *bli-3(im10)* also had a shortened lifespan on heat killed *E*. *coli* compared to wild type (*P* < 0.0001). The data are representative of experiments repeated three times with an n = 60–90 worms for each condition. All worms were exposed to *cdc-25*.*1* RNAi prior to beginning these assays to render them sterile.

The lesion in the peroxidase domain caused by the *bli-3(e767)* mutation did not increase *C*. *elegans* susceptibility to *E*. *faecalis*, despite the blistered cuticle phenotype. However, we postulated that susceptibility would be impacted by a mutation that affects the NADPH oxidase domain and ROS production [5]. As shown in Fig 3A and 3B, animals carrying such a mutation, *bli-3(im10)*, died more quickly on *E*. *faecalis* and *C*. *albicans* compared to wild type and *bli-3(e767)* strains. However, *bli-3(im10)* animals also expired more quickly than wild type when feeding on *E*. *coli* in a traditional lifespan assay (Fig 3C). Because *E*. *coli* can be slightly pathogenic [27], we also used heat-killed *E*. *coli*, but similar results were observed. These data suggest that loss of the NADPH oxidase activity affects a variety of functions that impact the health of these animals.

### BLI-3 is located in the hypodermis, the pharynx and the apical membrane of the intestine

**Fig 4:**
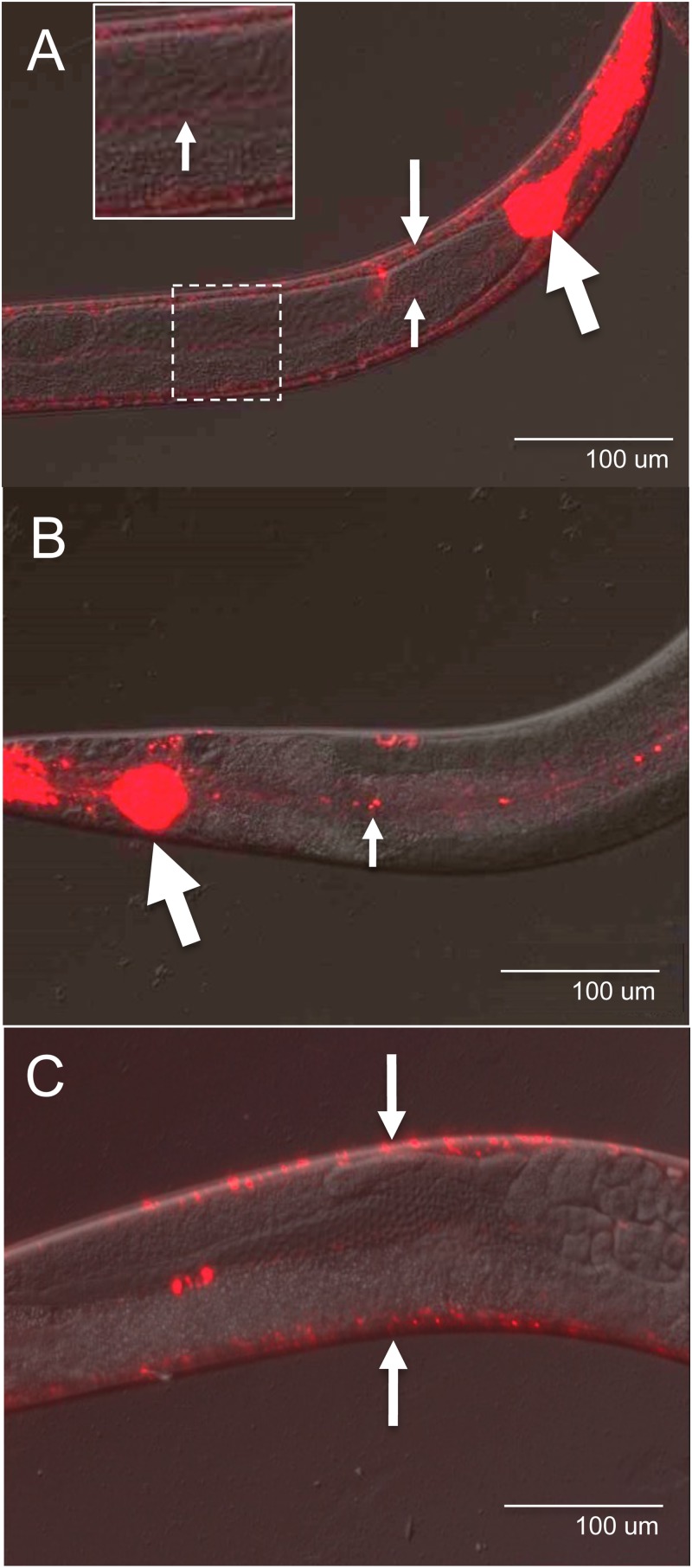
Localization of BLI-3 to the hypodermis, pharynx and apical membranes of the worm intestinal cells. **(A)** One of four transgenic lines showing expression of *BLI-3*::*mCherry* in the hypodermis (medium white arrow), pharynx (large white arrow) and the apical membranes of the intestinal cells (inset). **(B-C)** One of two integrated transgenic *BLI-3*::*mCherry* lines demonstrating expression in the **(B)** pharynx, apical membrane and the **(C)** hypodermis. Large white arrows indicate the pharynx, medium white arrows, the hypodermis, and the small white arrows, the apical membranes of the intestinal cells. Patterns of expression shown are typical for all animals of all lines.

Previous work employing a α-BLI-3 polyclonal antibody localized BLI-3 to the hypodermis. The hypodermis lies just under the cuticle and this pattern of localization is consistent with BLI-3’s function in cuticle cross-linking [8]. We postulated that BLI-3 might also be present in the intestine because intestinal-specific RNAi of BLI-3 resulted in susceptibility to *E*. *faecalis* [5]. To visualize BLI-3 using a different technique, we generated a plasmid in which the promoter region and first two exons of the gene were fused in-frame to *mCherry*. This plasmid was injected into *C*. *elegans* and the resulting transgenic lines were observed. An example of one of the four lines observed is shown in Fig 4A. In addition to the presence of fluorescent signal indicative of mCherry in the hypodermis, a strong signal in the pharynx and a weak signal in the apical membrane of the intestinal cells were also observed. Two of the lines were UV irradiated and backcrossed to select for stable, integrated transgenics. Examples from the two lines are shown in Fig 4B and 4C. While there were some differences in the intensity of the fluorescence, we still observed hypodermal, intestinal and pharyngeal fluorescence to some degree in all lines.

The presence of this protein in the apical membrane of the intestinal cells suggests that BLI-3 is located in this tissue like mammalian and Drosophila DUOX proteins [4,7]. The pharynx is known to contain cuticle, which connects to the cuticle in the epidermis, and it is therefore not surprising that BLI-3 would be located in this organ considering its crucial role in cuticle formation [28]. We postulate that localization to these internal tissues was not detected in the former study because the animals were not disrupted adequately during the fixation process to allow hybridization of the α-BLI-3 antibody to these internal tissues [8]. It is also possible that the transgenic lines display an altered expression pattern because of overexpression or lack of some more distant promoter element.

### Infection does not increase the amount of BLI-3 present

**Fig 5:**
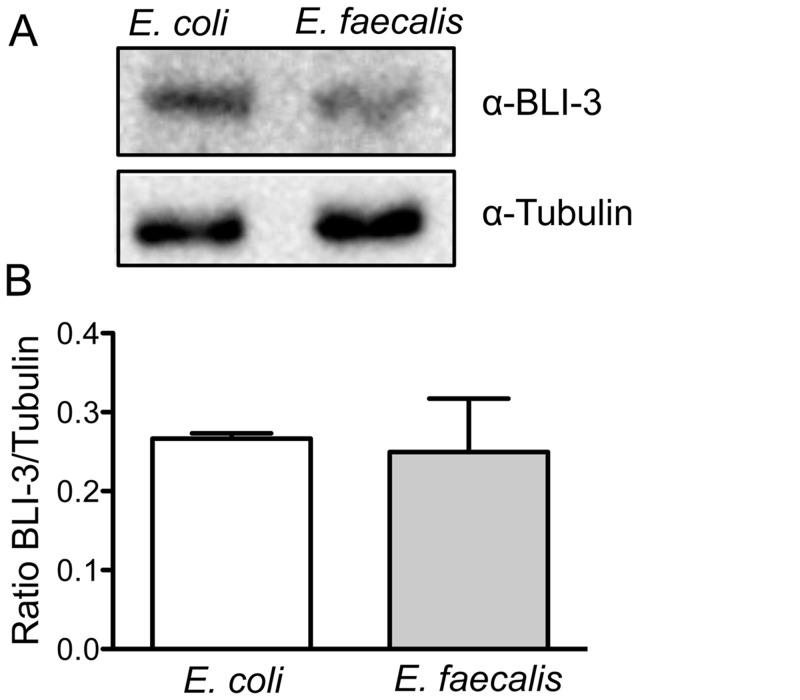
BLI-3 protein levels are similar in *E*. *faecalis* and *E*. *coli* fed worms. **(A)** A representative Western blot of BLI-3 protein expression in non-infected and infected worms. **(B)** For quantification of BLI-3, a ratio of BLI-3 protein to tubulin was calculated from independent experiments. The error bars represent the standard error of the mean.

In other systems, an increase in expression of the gene(s) encoding DUOX was associated with certain autoimmune conditions and pathologies (human) [29,30] and caused by certain types of infection (Drosophila) [7,31]. Various microarray studies have been performed on infected worms, but no increase in *bli-3* expression was noted, including those that examined *C*. *elegans* infected with *E*. *faecalis* [32,33]. When we exposed the stable, BLI-3::mCherry transgenic lines to *E*. *faecalis* no increase in the amount of fluorescence was observed (data not shown). To better quantitate the amount of BLI-3 protein present in infected vs. uninfected animals, we performed a western blot using anti-BLI-3 serum generated previously [8]. An example blot is shown in Fig 5A. Similar levels of BLI-3 were observed in animals feeding on *E*. *coli* compared to animals feeding on *E*. *faecalis*. Using quantification software, the amount of BLI-3 and tubulin (used as a control) was measured. The ratio of BLI-3 to tubulin was calculated, averaged between different experiments, and the results shown in Fig 5B. No significant difference in the amount of BLI-3 protein was observed. We conclude that in the worm, regulating the amount of protein present is not a mechanism used to control BLI-3 activity.

### Conclusions

From this study further characterizing the dual oxidase BLI-3 in *C*. *elegans*, we can conclude the following. The NADPH oxidase domain with its H_2_O_2_-generating activity is the source of the protective effects of BLI-3 during infection. This assertion is supported by our previous work showing a lack of phenotypes in strains containing mutations in the peroxidase domain and the identification of separate peroxidases with protective roles during infection with *E*. *faecalis* [5,34]. Additionally, we conclude that BLI-3 is generated in the pharynx in addition to the hypodermis and the intestine [5,8]. Infection does not stimulate further BLI-3 production or change the localization pattern.
